# European birth cohorts: a consideration of what they have addressed so far

**DOI:** 10.1186/s12887-022-03599-2

**Published:** 2022-09-15

**Authors:** Chiara Pandolfini, Rita Campi, Maurizio Bonati

**Affiliations:** Department of Public Health, Laboratory for Mother and Child Health, Istituto di Ricerche Farmacologiche Mario Negri IRCCS, Via Mario Negri 2, 20156 Milan, Italy

**Keywords:** Cohort studies, Data collection, Data sets as topic, Biomedical research, Europe, Longitudinal studies, Network analysis

## Abstract

**Background:**

Knowing the research issues addressed by other cohorts when setting up new cohorts allows researchers to avoid unnecessary duplication of efforts, while permitting collaborations, including data merging data, to better tackle knowledge gaps. This study describes the topics addressed by European birth cohorts, the interaction between these cohort interests and aims, and describes the scientific publications deriving from the cohorts.

**Methods:**

A previous study found 66 pregnancy and 45 birth cohorts in Europe. In this study, between August and October 2020, the predominant key areas addressed by the 45 birth cohorts identified in the previous study were evaluated, as were the publications found in PubMed that were associated with the 45 cohorts. A network analysis was performed to show the connections between the 13 key areas identified. A focus on a topic in common between two areas was provided, describing the related publications.

**Results:**

A total of 1512 references were found in PubMed (148 publications per cohort). Thirteen predominant key areas were identified, the most common of which was “Environmental” (addressed by 20 cohorts). The Environmental, Genes, and Lifestyle exposure areas were the prevalent topics characterizing the network figure. The Environmental area had the largest number of interactions with the other areas, while the Prematurity area (4 cohorts) the least. The focus provided on smoking led to the comparison of 35 publications from the Environmental group of cohorts and 22 from the Prematurity group, but their objectives did not overlap.

**Conclusions:**

The results of this descriptive study show that the environment is a priority research area for cohorts in Europe and that cohorts with different research areas may have study issues in common, but may approach them from different viewpoints. Birth cohorts have wide-ranging aims and it would be almost impossible, and undesirable, to have perfectly overlapping and comparable objectives, but joining efforts would permit maximum use of available resources.

**Supplementary Information:**

The online version contains supplementary material available at 10.1186/s12887-022-03599-2.

## Background

Cohort studies are a type of observational research design used to investigate the causes of disease and determine links between risk factors and health outcomes. Birth and pregnancy cohorts, in particular, are useful tools for studying the early origins of health and the emergence of diseases. Pregnancy cohorts usually begin in pregnancy, focus on the pregnancy, and sometimes continue after birth. Birth cohorts begin at birth and focus on the child, sometimes collecting data on the pregnancy retrospectively to identify maternal or pregnancy-related risk factors. Prospective, population-based cohorts are capable of collecting a vast array of data and of providing long term follow-ups, even lifelong ones. These characteristics permit them to identify possible links between early exposures (in-utero or perinatal) and health outcomes later in life. This field of research, and the consequent, related knowledge, has been expanding over the last few decades. Specifically, epigenetics, which refers to the mechanisms that result in phenotypic trait differences caused by environmental factors that impact on gene regulation, has been described as the potential mechanism behind development programming in the context of DOHaD, the developmental origins of health and disease [1]. The DOHaD concept is centered around the fact that a vast array of environmental factors, as well as the interactions between environmental and genetic factors, both in-utero and in the perinatal period, influence health and risk of disease in later life [2].

Numerous birth cohorts have been set up worldwide [3–6]. One of the oldest cohorts began in the UK in 1921 [7] and was set up to study the effects of childhood mental ability on survival later on in life, while more recent cohorts, such as the KUNO cohort [8], tend to study broad areas that can encompass any factors influencing child health and opportunities for prevention. The scope of the aims of cohorts has, in fact, expanded over time, as has the amount of data collected. This is likely also due to the increasing awareness of the DOHaD.

The characteristics of pregnancy and birth cohorts, and the general research areas they were designed to address, have been described in different reviews [5, 9, 10]. The more common areas covered are immune disorders (allergic diseases and asthma), environmental exposure, neurocognitive development/neurobehavioral disorders, and nutrition. To our knowledge, however, only two reviews have reported the research areas of cohorts with general aims, i.e., covering all aspects of child development and health [5, 9], and these reviews simply listed the cohorts’ overall study areas. A 2017 study also attempted to assess the areas covered by the scientific publications deriving from cohorts [11]. This study analyzed the publications deriving from 20 cohorts that addressed the environment and noncommunicable diseases specifically, as a proxy for a cohort’s scientific potential, and showed the subject categories of the journals in which the articles had been published [11]. The research areas of the articles were not analyzed directly, however, so the areas reported are broader and not comparable with other research.

Knowing the research issues addressed by other cohorts when setting up new cohorts, and deciding their objectives, is important because it permits the new cohorts to be designed in such a way as to add to scientific knowledge and avoid waste of valuable resources, as often occurs with clinical trials [12]. More specifically, it can assist in avoiding duplication of efforts, such as in carrying out similar cohorts in populations that are have the same geographic, age, and socio-economic setting characteristics, without ensuring that variables or the data collection method are similar and comparable with those of other cohorts. At the same time, it can help replicate the necessary aspects, as in collecting similar data in different populations, in order to be able to collaborate or merge data and to address areas characterized by knowledge gaps [13]. In this context, starting from a 2020 review of European pregnancy and birth cohorts [5] published by us that provided a general description of the cohorts and a focus on the 45 birth cohorts, we decided to analyze the predominant key areas addressed by these birth cohorts. Our aim was to identify the topics addressed and the interaction between these cohort interests and aims, assess which areas were studied individually or in collaborative studies between cohorts, and describe the scientific publications deriving from these cohorts.

## Methods

A 2020 review [5] identified 111 European cohorts (66 pregnancy and 45 birth cohorts) and provided a brief description of them, also identifying the key scientific areas they addressed, based on their title and declared aims. The full methodology used to identify and select the cohorts was described in the 2020 publication [5], but, briefly, the Medline (PubMed) and Embase databases were searched for articles referring to longitudinal, prospective European cohorts that started recruitment in pregnancy or at birth (referred to as pregnancy or birth cohorts, respectively), excluding randomized controlled trials and articles focusing on vaccines or on genes or gene expression. Online cohort inventories were searched as well. The records found in the two databases were then reviewed and the name of the cohort the articles involved was noted. In this study, we evaluated the predominant key areas addressed by the set of 45 birth cohorts found in the 2020 study, assessed publications deriving from these cohorts, and selected an example on which to provide a focus with a more thorough description. We also performed a network analysis to describe the relationship between the different areas addressed by the cohorts. We excluded the 66 pregnancy cohorts, among which were two of the largest pregnancy cohorts, the Norwegian Mother Father and Child cohort and the Danish National Birth Cohort, in order to limit the number of cohorts and to be able to assess the already numerous cohort publications found.

Specifically, between August and October 2020, an author independently searched PubMed for all publications deriving from each of the 45 cohorts, modifying the search string several times to maximize sensitivity/specificity. Search strings varied greatly based on cohort name (i.e., if the cohort’s name/acronym was also a common term instead of a unique one, such as for the Prenatal Cohort, it was not possible to simply search for the cohort name). Attempts to limit irrelevant results from PubMed were made by excluding articles published prior to each cohorts’ enrolment start date. Additional articles were found through article citations and were added to the database. The search strings and all publication limitations applied are listed in Additional file 1.

The bibliographical records, including the abstracts, medical subject headings (MeSH terms), and author keywords resulting from the searches were downloaded into the Zotero program (www.zotero.org; a free reference management software to manage bibliographic data and related research materials), applying a tag with the name of the cohort to each set of references found. An author then independently checked the Zotero database to further exclude references if they only mentioned the cohort name, but did not report data on that cohort, if they referred to a later batch of recruitment of the same cohort (e.g., Slovakia PRENATAL cohort), or if they were corrigendums to existing articles.

Duplicate publications were checked and, if the article referred to multiple cohorts, one copy of the article for each cohort was kept in the database, with the accompanying cohort name tag.

In 34 records the MeSH terms were not present, so the records of those articles were searched for in the EMBASE database and their related medical subject headings (EMTREE terms) were copied into the Zotero database. If the records were not present in EMBASE, the online full text version of the article was found, if accessible, and the author keywords were used, when available.

The database, including the keywords (MeSH terms plus author keywords), was then exported to Microsoft Access. In an attempt to clean the keywords and to omit at least a part of the irrelevant ones, two authors jointly reviewed all terms present at least twice and reclassified them when necessary, e.g., when the same term was written in different ways (“breast feeding” and “breastfeeding”), or when synonyms were present. Furthermore, keywords referring to a place (e.g. Europe), a cohort’s name (e.g. BAMSE), or an age group (e.g. child) were deleted because they were not relevant to the scientific area addressed by the publications.

The key scientific areas of the 45 cohorts, as originally listed in the 2020 publication, were used, but were slightly modified to make them more specific. To do this, for the five cohorts originally reported as having “multiple aims”, the specific, individual aims were now listed, while for all the cohorts, only the predominant key areas were kept. We identified as environmental area cohorts those that performed research on environmental exposures such as air pollution or maternal tobacco smoke, and identified as prematurity area cohorts those whose declared study areas referred to the term premature/prematurity, even if their criteria varied somewhat from the general definition that involves babies born before 37 weeks of pregnancy. This led to 13 key areas.

A network analysis was performed to show the relationship between the key areas. The Fruchterman-Reingold algorithm, which uses an iterative process to adjust the placement of the nodes (in this case “areas”) in order to minimize the energy of the system, was implemented [14]. At the core of the network are the nodes with high associations, while the nodes with low associations are in the peripheral areas. The figure generated shows the most consistent associations, where thicker edges show stronger relationships and thinner edges weaker relationships. In order to qualify the importance of each node in the relative importance network, we used three indices of centrality: betweenness, closeness, and Eigen [15]. Betweenness centrality measures all the shortest paths between every pair of nodes of the network and then counts how many times a node is on a shortest path between two others. Closeness centrality is a measure of the proximity of a selected node to all other nodes within the graph. Eigen centrality measures a node’s influence based on the number of links it has to other nodes in the network and can identify nodes with influence over the whole network, not just those directly connected to it. The comparison of centrality score is used as a measure of nodal roles in networks to indicate the influence of each centrality measure on the nodes on the network.

A community detection analysis was carried out in order to better understand the functionality of the network. Community detection analysis is used to discover and identify communities in the network by creating clusters of the different nodes that have potentially similar characteristics. Louvain’s algorithm was used [16] to optimize modularity, which measures the relative density of edges inside communities with respect to edges outside communities, leading to the best possible groupings of nodes given a network [17]. Gephi software was used for all network analyses [18].

The areas least and most connected with other research areas (in terms of number of interactions represented in the network analysis), and their objectives, were then further assessed.

We then selected a keyword from among the most frequent keywords in common from the publications of the cohorts from the two selected areas (Environmental and Prematurity), and provided a brief focus on that topic. Specifically, the publications indexed with that selected keyword were then identified and the individual issues they addressed were extracted from the titles and abstracts.

We then wanted to see if there was an overlap between the publications of the cohorts from the two areas, representing interaction and collaboration of cohorts, and searched the keywords of the publications for each area for the presence of terms related to the other area.

## Results

A total of 1750 references of publications were found from the PubMed searches. Once the non-pertinent references were deleted (mostly because: they only mentioned the cohort name, but were not relevant to the cohort; because the use of a cohort’s name in the search string led to irrelevant results, e.g. “Epicure” is also a software package; or because they were corrigendums to existing articles on a cohort), 1512 records remained. The number of publications ranged from 1 (for 3 cohorts) to 148 (1 cohort), with a median of 17 publications per cohort. Publication dates ranged from 1981 to 2021 (median 2015), with 81% of articles published from 2010 onwards. The cohort dates and status contribute to the number of publications, in that many cohorts are closed, while 4 are still enrolling and 21 are in the follow-up phase.

A total of 24,662 keywords were present in the 1512 references and, after reclassification and exclusion of many nonrelevant ones, 16,741 remained.

The predominant key areas of the 45 cohorts are reported in Table 1. A list of the main objectives of the cohorts and a reference to each cohort is available in the 2020 review cited above. A total of 13 predominant areas was identified, the most common of which was the “Environmental” area (addressed by 20 cohorts), followed by “Allergic diseases” (15 cohorts), and “Growth” and “Lifestyle exposure” (10 each). Individual cohorts addressed from 1 (13 cohorts) to 5 (2 cohorts) of the predominant areas identified. One half of the cohorts (22 cohorts) addressed 3 or more areas.

**Table 1:** List of the 45 birth cohorts and the predominant key areas involved for each

Cohort	Country	Enrolment start date	Follow-up ongoing	Environmental	Neurocognitive development	Growth	Allergic diseases	Prematurity	Respiratory	Health	Asthma	Lifestyle exposure	Nutrition	Twins	Obesity	Genes
ABERDEEN	UK	1921			X					X						
ABIS	Sweden	1997		X			X				X					X
ADAPAR	Turkey	2010					X									
AUBE	France	2009			X											
BAMSE	Sweden	1994	Yes				X				X	X				
CCC2000	Denmark	2000			X											
CZECH	Czech Republic	1994		X					X							
DARC	Denmark	1998					X									
DONALD	Germany	1985	Yes			X				X			X			
DUTCH1990	The Netherlands	1990			X										X	
ECA	Norway	1992		X							X					
ELFE	France	2011	Yes	X						X		X				
ENVIRONAGE	Belgium	2010	Yes	X								X				
EPICURE1995	UK	1995	Yes					X								
EPIFANE	France	2012											X			
EPIPAGE	France	2011	Yes			X		X								
EUROPREVALL	Multicenter	2005					X									
FAIRCOHORT	UK	2001					X									
FLEHSI	Belgium	2002		X												
G21	Portugal	2005	Yes			X										
GASPII	Italy	2003	Yes	X						X		X				X
GEMINI	UK	2007	Yes	X		X								X		X
GINIPLUS	Germany	1995	Yes		X		X		X		X		X			
GMS	UK	1999	Yes			X							X			
GUS	UK	2004	Yes	X	X							X				X
HALLAND	Sweden	2007				X										
HUMIS	Norway	2003	Yes	X												
ITALNEONAT	Italy	2009						X	X							
KUNO	Germany	2015	Yes	X			X			X		X	X			
LISAPLUS	Germany	1997		X			X					X				X
LRC	UK	1985	Yes						X							
LucKi	The Netherlands	2006	Yes				X								X	
MAS90	Germany	1990		X			X					X				
MUBICOS	Italy	2009	Yes	X										X		X
NFBC1986	Finland	1985				X		X		X						
PARISCOHORT	France	2003		X			X		X			X				
PCBCOHORT	Slovakia	2002		X	X		X									
PICCOLIPIU	Italy	2011	Yes	X						X		X				X
PRENATAL COHORT	Slovakia	1997		X			X						X			
TEDS	UK	1994	Yes	X	X									X		X
TERNEUZEN	The Netherlands	1977				X									X	
TURKU1981	Finland	1981			X	X			X							
UBCS	Germany	2000				X				X					X	
ULMSPATZ	Germany	2012	Yes				X				X				X	
WHISTLER	The Netherlands	2003	Yes	X					X							X

**Fig. 1:**
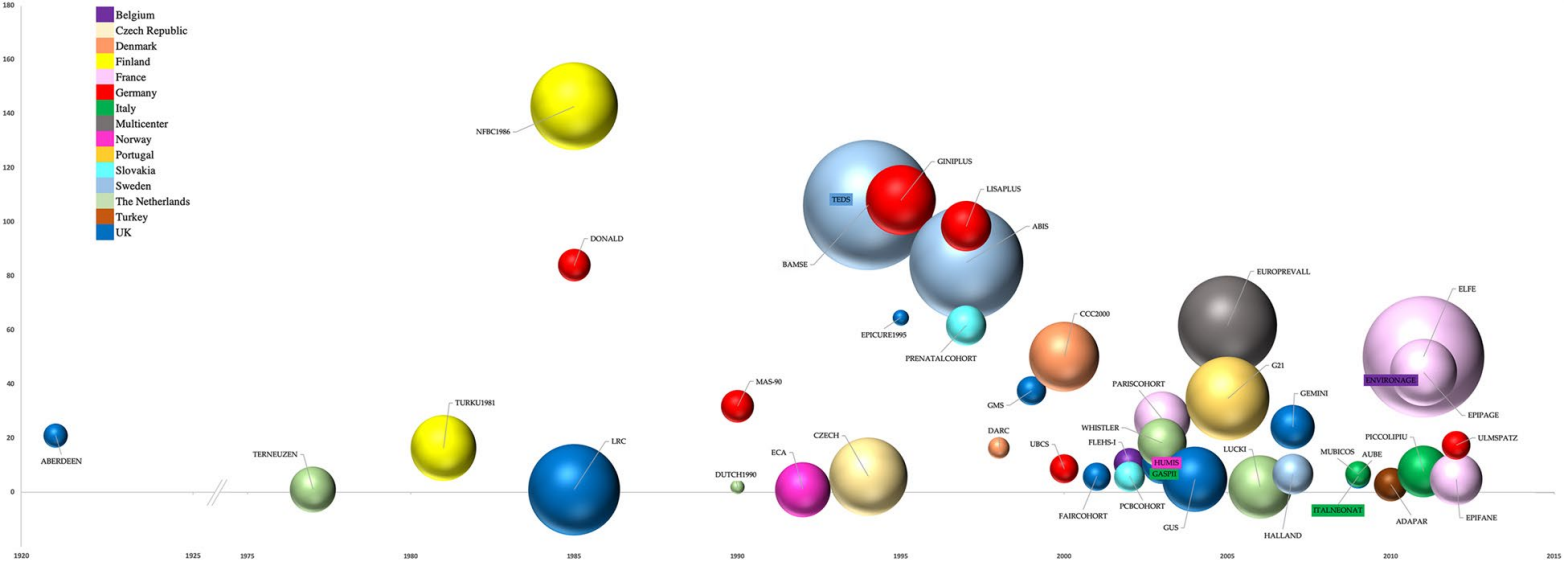
Number of publications (y-axis) by starting year of cohort (x-axis) (size reflects population size, color reflects country of cohort)

Figure 1 shows the distribution of the number of publications by starting year of the cohorts. The size of the spheres represents population size and color represents the countries of the cohorts.

**Fig. 2:**
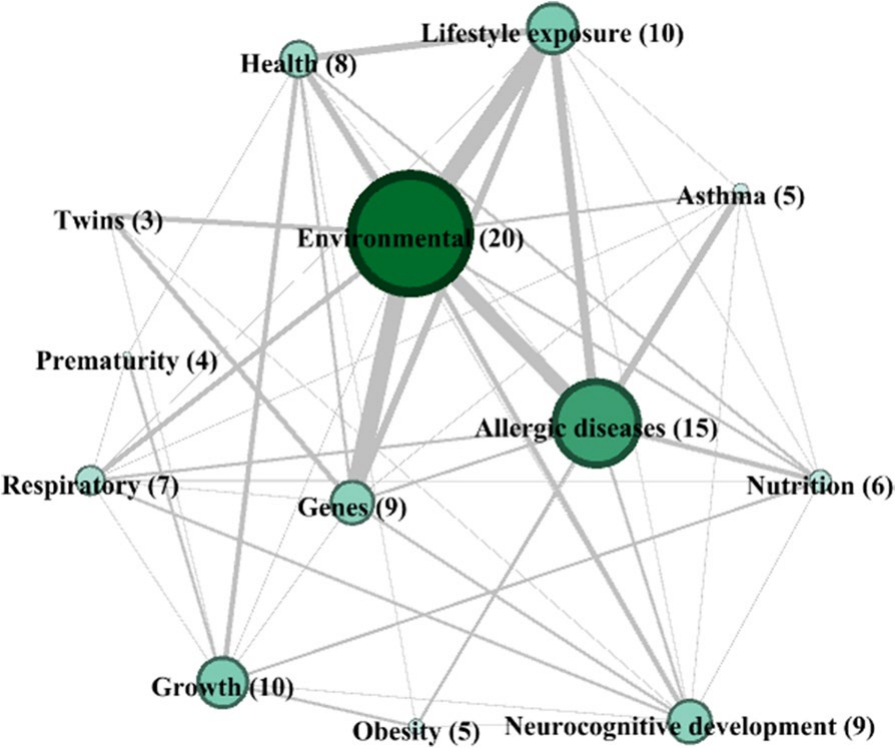
Network analysis of the 13 predominant key areas of the 45 birth cohorts (number of cohorts addressing that area)

The predominant key areas among the cohorts’ aims, and the relationships between the different areas, are shown in the network structure in Fig. 2. The diameter of the nodes refers to the degree of centrality and the hue of the node refers to betweenness centrality (darker = higher value). The weights of the connections are presented in Additional file 2. Strong connections emerged among environmental and lifestyle exposure, among environmental and genes, and environmental and allergic diseases, as shown clearly in the structure of the network (Fig. 2).

The community detection analysis identified 3 clusters, shown in Additional file 3. One cluster was formed by the Environmental, Health, Lifestyle exposure, Twins, and Genes areas, a second cluster by Neurocognitive development, Allergic diseases, Respiratory, Asthma, Nutrition, and Obesity, and a third by Growth and Prematurity.

Lastly, the centrality indices are shown in Additional file 4. The three indices are significantly intercorrelated with each other: the correlation between eigen and closeness is 0.96 (*p* < 0.01), the correlation between eigen and betweenness is 0.59 (*p* = 0.03), the correlation between closeness and betweenness is 0.74 (*p* = 0.003). The resulting plot for centrality metrics highlights the differences in the connectivity of the network. The Neurocognitive area has the most connections in the network, but with weak relationships with the other nodes, while Growth ranks the highest on betweenness and acts most often as a bridge on the shortest path between two other nodes. The closeness and eigen measures show that Environmental, in addition to Neurocognitive development, have higher centrality measures.

All centrality measures indicate that Prematurity and Twins are the least central nodes. Nutrition, Lifestyle exposure, Asthma, Health, and Growth are the most central variables, considering the eigen measure, so these nodes might not be thought of as important on their own, but their relationship to other highly connected nodes indicates a high level of influence.

The Environmental area has the largest number of interactions with the other areas, while the Prematurity area has the least. These two areas were therefore chosen as examples. The enrolment starting dates for the 20 Environmental area cohorts ranged from 1990 to 2015, and, as of October 2021, 10 had currently ongoing follow-ups. A total of 629 articles on these 20 cohorts had been published. The Prematurity area, on the other hand, is represented by 4 cohorts, their enrolment starting dates ranged from 1985 to 2011, and 2 had currently ongoing follow-ups. A total of 267 papers had been published by these 4 cohorts. None of these publications involved cohorts belonging to both groups, i.e. cohorts with both Environmental and Prematurity predominant areas.

A focus on smoking is provided because smoking was among the most frequent, shared topics between the publications on the Environmental and Prematurity areas. Additional file 5 lists the 35 publications concerning smoking that originated from the Environmental area cohorts (12 of the 20 cohorts are represented by these publications), and the 22 publications from 3 of the 4 Prematurity area cohorts. The topics of these 57 publications varied widely, and a few topics were in common between the two groups, such as birth weight, asthma, and body mass index (BMI). In a few publications, smoking was considered indirectly, for example as a covariate. Of the 35 Environmental area cohort publications involving smoking, two looked at the outcome of the effects of direct exposure to smoking in adolescents, while the rest looked at the effects of maternal/paternal smoking (mostly the effects of indirect exposure on long-term outcomes in offspring). In the prematurity area group of cohorts, of the publications looking specifically at prematurity, none looked at the outcome of the effects of direct exposure to smoking in newborns/children, and one looked at the outcome of indirect exposure, studying how prenatal determinants are associated with physical activity and cardiorespiratory fitness in adolescence [19]. None of the publications from the Environmental group addressed indirect exposure on physical activity or cardiorespiratory fitness. To further attempt to see if there was an overlap between the Environmental and the Prematurity area cohort publications, all 629 Environmental cohort publications were searched for the keywords “infant, premature” or “preterm birth”, and three publications were found [9, 20, 21]. These looked at inflammatory bowel disease in parents and neonatal outcome, and extremely low frequency electromagnetic fields and the risk of prematurity (2 publications). On the other hand, 2/267 Prematurity area cohort publications looked directly at prematurity and had the keyword “environment”: one looked at the effects of traffic on the risk of hospitalization for bronchiolitis [22] and the other looked at the effects of the social environment on preterm delivery [23].

## Discussion

This study is not a systematic review. The network analysis approach can be used in support of the standard review method, and, in this case, was used to analyze the general study areas of the cohorts and to compare similar or differing cohorts in terms of aims, and to observe the interactions between them. This kind of approach produces an outlook on overlapping research areas, and was integrated with a comparison of published articles, based on the keywords they were labelled with, to further describe the cohorts’ aims and research areas.

The attempt to identify the prevalent topics and interests of cohorts is useful to help steer research towards knowledge gaps and to promote collaboration between cohorts. The environment, and its effects on children’s health, was found to be the most commonly studied topic by the 45 cohorts. Environmental factors, such as ambient pollution, have increasingly been studied over the years. The last twenty years have seen a rapid increase in the study of effects, at lower doses, of a more diverse assortment of environmental agents, such as industrial agents and endocrine disruptors, on health, growth and development of children [24]. Allergic diseases were the next most common area and this, too, reflects the increasing interest in this field in the last decades, likely due to the increasing prevalence of allergies [25]. It is interesting to see which research areas addressed by cohorts so far are “isolated” and which are most connected with other areas. Among the European birth cohorts assessed in this study, research on prematurity seemed to be the narrowest, restricted to more specific topics, as shown by the network analysis.

The clusters identified in the community detection analysis reflect areas that are connected in terms of comorbidities or covariables related to interactions in the physiological and pathological aspects of health.

The publications of the cohorts from the two areas chosen as examples were looked at more thoroughly, and the research topics addressed hardly overlapped, even if the data collected and the variables analyzed may in part have been the same. None of the publications involved cohorts belonging to both groups, i.e. cohorts with Environmental and Prematurity predominant key areas, and this supports the results of the network analysis, which show that the two groups of birth cohorts do not overlap in terms of their main research areas. From the Environmental group of cohorts, the three publications that looked at prematurity looked only inflammatory bowel disease in parents [26] or the effects of electromagnetic fields [27, 28]. Of the 2 Prematurity cohort publications that looked at environment, one looked at environmental exposure in terms of exposure to substances [29], while the other looked at the effects of the social environment [30]. The publication dates of the Environmental area cohort articles ranged from 1981 to 2020 (80% of which ≥2010), while those of the Prematurity area from 1995 to 2020 (82% of which ≥2010), so they overlap in terms of time periods.

In general, the birth cohorts taken into consideration in this study, and their publications, cover an extensive time range, however, with many cohorts that are closed and others that are currently ongoing. These differences in time periods limit a direct comparison of topics, as well as potential collaboration between cohorts with similar aims. This issue does not necessarily have to preclude collaboration between closed and ongoing cohorts that may profit from joining forces, however, since closed cohorts could still make their data available for joint analyses.

When smoking, one of the most common topics in common between the publications of the two groups of cohorts, was considered, the studies did not have the same objectives. Most of the publications from the Environmental cohort group looked at the outcomes of indirect exposure, i.e., long-term maternal/paternal effects of smoking in the offspring, while, of the publications looking specifically at prematurity from the other group, only one looked at the long-term (indirect) outcomes. In general, there does not seem to be a tangible connection in terms of research areas between the two groups, even though common research topics exist. Two of the prematurity cohorts are currently still following-up their participants, as are half of the environmental cohorts, so, if one considers the smoking example, it would be possible to attempt to collaborate by deciding on specific, shared data to collect at follow-up and to merge results and exchange viewpoints.

There are likely numerous areas such as this one in which an effective network or collaboration would be advantageous. Birth cohorts have wide-ranging aims and it would be almost impossible, and undesirable, to have perfectly overlapping and comparable objectives (some cohorts are purposely nonrepresentative of the general population so as to emphasize contrast between environmental exposures) [31], but joining efforts and resources would permit researchers to fill gaps in knowledge and maximize use of limited available resources, given the costly and resource-consuming characteristic of cohorts. Even more importantly, collaborating would permit pooling analyses to increase statistical power, joining different types of data based on each cohorts’ collection methods and capabilities, and comparing different geographic or cultural contexts, all of which would increase the power of inferences [9]. This joining of efforts could entail working together side by side or merging data afterwards, such as for the MeDALL project [32], a European research initiative on asthma and allergy that merges data from multiple cohorts. A European-wide cohort survey is also being set up that will follow-up children for 24 years [33]. It would be interesting, for the future, to set up a new network of cohorts or other research studies applying the European burden of disease definitions to the data collected. Some research has already begun in this regard, in fact, and has attempted to quantify the long-term outcomes of maternal and infant interventions to prevent obesity of chronic diseases [34], and the lifelong health impacts of individual risk factors such as maternal smoking [35]. More efforts should, in any case, be devoted to collaborative and multinational convergences.

In this context, efforts are underway internationally to join efforts in researching common issues and in joining data from different cohorts into a common data repository [36].

There is a fine line between similar and duplicate cohorts. Cohorts that collect similar data on comparable populations produce useful data that can be merged, while duplicate cohorts produce redundant data (e.g. data on the same population) and represent a waste of resources. If one considers prospective cohorts, this waste of resources increases continuously over time. Avoiding the duplication of research efforts is difficult [37]. Descriptions of existing cohorts, such as this one, or initiatives such as birthcohorts.net, which provide a searchable list of general data from different existing cohorts, can help researchers shape future cohorts so that they do not represent unnecessary duplicate efforts. Analyses of the research areas addressed by cohorts, such as the one described in this article, should be carried out on a larger, broader scale, and updated regularly so that they can be useful for new and ongoing cohorts, worldwide.

In this context, as with clinical trials, systems to uniquely identify cohorts, and their publications, would facilitate researchers wishing to report on cohorts or synthesize or compare their published data without including repeat information. In fact, cohorts change over time (population numbers, reopening of enrollment in time, use of different sub-cohorts in different studies, new batches of subjects enrolled at once and added to the cohorts, etc.) and this leads to apparently differing descriptions of the same cohort, and its population, in different articles. The lack of a national or international identification number identifying a cohort and its publications therefore often makes it difficult to match data reported in the published literature with the cohort of origin, unless a cohort has an original, unmistakable name that is cited clearly in all publications.

Many of the ongoing cohorts are surely already studying the effects of the current COVID-19 pandemic in all their facets, from psychological ones to the long-term health-related effects of exposure to the SARS-COV2 virus and its related vaccinations. A positive characteristic of some cohorts is, in fact, the ability to adapt to respond quickly to emerging research questions even if not initially contemplated. It seems that larger cohorts with more general aims, collecting background information in addition to more specific data on the population from multiple sources, including biological samples, may be more capable of adapting, as well as of addressing long-term research, and therefore of influencing research policy in general [11].

This study has strengths and limitations. The lack of a worldwide, acknowledged registration number for cohort studies makes it challenging to identify cohorts and their related publications in the biomedical literature databases. The strength of this study is its originality in that attempts were made to search for all publications related to the cohorts with distinct, customized search strings for each cohort, in order to best identify their prevalent topics and interests. The first of the three limits of this study is that it was not possible to know if all publications related to each cohort were found. Despite efforts to search for all related publications, some may not have been identified, those identified may not have been attributed to all participating cohorts, or others may have been attributed erroneously to a cohort, for the reasons explained previously. The second limit is that, given the broad nature of cohorts in terms of the areas studied makes the cohorts’ aims difficult to classify. The overall set of keywords with which the cohort-related publications were indexed was considerable and wide-ranging, and it was therefore difficult, despite an attempt to clean the keywords, to use the terms to describe the topics addressed by the publications and, consequently, to describe the cohorts’ general aims. Along the same lines, the aims of the cohorts can be very broad and can cover vast research areas. The aims may even evolve over time. The predominant key areas identified are therefore only generally representative of the cohorts’ aims. The third limit is that this study assessed birth cohorts, not pregnancy cohorts, and the research areas of birth cohorts may not entirely reflect those of pregnancy cohorts.

## Conclusions

This study found that the environment was the most commonly studied topic by the 45 birth cohorts, and that it was the area that was studied together with the largest number of other areas. Prematurity, on the other hand, was found to be the area studied most independently, i.e., the research on prematurity was restricted to more specific topics. Furthermore, the results of this descriptive study show that cohorts with different main research areas may have study issues in common, but may approach them from different points of view. It is well known that by joining efforts and resources cohorts can fill gaps in knowledge more easily and maximize the use of available resources, and studies such as this one may help identify areas in which ongoing or future cohorts can invest and can collaborate even if their general objectives are different.

Continuing to monitor ongoing cohorts is necessary to, both, promote more efficient investments in research efforts, and to direct these efforts towards where the greatest knowledge gaps are. Monitoring cohorts also permits the identification of research areas that could benefit if studied with more adequate, commonly acknowledged, or standardized, methodologies.

## Supplementary Information

**Additional file 1.**

**Additional file 2.**

**Additional file 3.**

**Additional file 4.**

**Additional file 5.**

**Additional file 6.**

**Additional file 7.**

## Data Availability

The datasets supporting the conclusions of this article are included within the article and its additional files.

## Abbreviations

BMI: Body mass index
DOHaD: The developmental origins of health and disease
MeSH: Medical subject headings
