# Supplementary material for: European birth cohorts: a consideration of what they have addressed so far
Source: BMC Pediatr. 2022 Sep 15;22:548. doi: 10.1186/s12887-022-03599-2 (PMC9476293; doi:10.1186/s12887-022-03599-2)
Supplement: Supplementary file 1 — Additional file 1. [file 12887_2022_3599_MOESM1_ESM.docx]

**Additional file 1.** References related to the 45 cohorts and the search strings used to find each cohort’s publications in PubMed.

| Cohort | Enrolment start | Search string | Limits applied to search string or search results | N publications | Dates of publications |
| --- | --- | --- | --- | --- | --- |
| **ABERDEEN**[1] | 1921 | Aberdeen AND 1921 AND cohort |  | 21 | 1999-2020 |
| **ABIS**[2] | 1997 | ABIS[Title/Abstract] AND (cohort OR study) AND Sweden |  | 88 | 1999-2020 |
| **ADAPAR**[3] | 2010 | Adapar[Title/Abstract] |  | 3 | 2015-2020 |
| **AuBE**[4] | 2009 | AuBE[Title/Abstract] AND (cohort OR study) AND (France OR French) | Publication year | 7 | 2009-2020 |
| **BAMSE**[5] | 1994 | BAMSE AND cohort AND Sweden |  | 110 | 2002-2020 |
| **CCC2000**[6] | 2000 | (CCC2000 OR CCCC2000) OR ("Copenhagen Child Cohort") OR ("Copenhagen County Child Cohort") |  | 52 | 2005-2020 |
| **CzECH**[7] | 1994 | Czech AND Early Childhood Health study AND 1994 |  | 6 | 2001-2020 |
| **DARC**[8] | 1998 | ("DARC" AND Denmark AND (cohort OR study)) NOT "Danish National Research Foundation Centre for Cardiac Arrhythmia" |  | 17 | 2005-2020 |
| **DONALD**[9] | 1985 | ("DONALD study" OR "Dortmund Nutritional and Anthropometric Longitudinally Designed study") AND Cohort AND Germany |  | 87 | 1998-2020 |
| **Dutch**[10] | 1990 | ("Dutch Cohort" OR "dutch birth cohort") AND Netherlands AND 1990 |  | 2 | 2011 |
| **ECA**[11] | 1992 | "environment and childhood asthma" AND Oslo |  | 1 | 2002 |
| **ELFE**[12] | 2011 | ELFE[Title/Abstract] AND (cohort OR study) |  | 52 | 2011-2020 |
| **ENVIRONAGE**[13] | 2010 | ENVIRONAGE[Title/Abstract] AND (cohort OR study) |  | 43 | 2013-2020 |
| **EPICure**[14] | 1995 | Epicure[Title/Abstract] AND (cohort OR study) |  | 67 | 1995-2020 |
| **Epifane**[15] | 2012 | Epifane[Title/Abstract] |  | 5 | 2016-2020 |
| **EPIPAGE 2**[16] | 2011 | EPIPAGE 2[Title/Abstract] AND (cohort OR study) |  | 46 | 2014-2020 |
| **Europrevall**[17] | 2005 | Europrevall AND (cohort or study) |  | 64 | 2007-2020 |
| **FAIR**[18] | 2001 | "Food Allergy and Intolerance Research" |  | 6 | 2015-2020 |
| **FLEHS-I**[19] | 2002 | FLEHS AND Flemish Environment and Health Study AND 2002 |  | 11 | 2002-2020 |
| **G21**[20] | 2005 | (((G21 AND (cohort or study)) OR "Generation XXI") AND Portugal[Title/Abstract]) |  | 36 | 2013-2020 |
| **GASPII**[21] | 2003 | GASPII OR Gene And Environment Prospective Study Of Infancy In Italy |  | 8 | 2007-2020 |
| **GEMINI**[22] | 2007 | GEMINI AND twins AND (cohort OR study) | Publication year | 25 | 2007-2020 |
| **GINIplus**[23] | 1995 | GINIplus |  | 112 | 2008-2020 |
| **GMS**[24] | 1999 | Gateshead millennium study |  | 39 | 2006-2020 |
| **GUS**[25] | 2004 | GUS AND Growing Up in Scotland |  | 5 | 2012-2020 |
| **H2GS**[26] | 2007 | ((Halland Health and Growth Study AND Sweden) OR (south-western Sweden[Title/Abstract] OR south-west Sweden[Title/Abstract]) AND cohort) AND Halland |  | 7 | 2011-2020 |
| **HUMIS**[27] | 2003 | HUMIS AND (Norway Or Norwegian) AND (cohort OR study) | Publication year | 11 | 2009-2020 |
| **ITAL NEONAT**[28] | 2009 | ("Multicenter Italian Birth cohort" AND (2009:2020[pdat])) AND ("Respiratory Tract Infections"[Mesh]) | Publication year | 6 | 2009-2020 |
| **KUNO**[29] | 2015 | KUNO Kids birth cohort |  | 2 | 2019-2020 |
| **LISA PLUS**[30] | 1997 | (LISA-plus OR LISAplus) AND Germany |  | 102 | 2006-2020 |
| **LRC**[31] | 1985 | LRC AND (Leicestershire OR Leicester) AND cohort |  | 1 | 2018 |
| **LucKi**[32] | 2006 | LucKi AND (Netherlands OR Dutch) |  | 2 | 2014-2020 |
| **MAS-90**[33] | 1990 | (MAS-90 OR MAS90 OR "Multizentrische Allergie studie" OR "Multicentre Allergy Study") AND cohort |  | 33 | 1994-2020 |
| **MUBICOS**[34] | 2009 | ("Multiple Birth Cohort Study" OR "Multiple Births Cohort Study") AND Italy AND twins AND Brescianini | Publication year, author name | 7 | 2009-2020 |
| **NFBC8586**[35] | 1985 | "Northern Finland Birth Cohort" AND 1986 | Publication year | 148 | 1997-2020 |
| **PARIS**[36] | 2003 | PARIS AND (Pollution and Asthma Risk: an Infant Study OR "Paris birth cohort") AND (cohort OR study) | Publication year | 28 | 2003-2020 |
| **PCB**[37] | 2002 | (Slovakia AND ("PCB Cohort" OR Early Childhood Development and PCB exposure in Slovakia)) OR (Slovakia AND ("PCB Cohort" OR Early Childhood Development and PCB exposure in Slovakia OR Slovak PCB Effects on Early Child Development Study)) |  | 6 | 2006-2020 |
| **Piccolipiù**[38] | 2011 | Piccolipiu |  | 8 | 2014-2020 |
| **PRENATAL**[39] | 1997 (1996-2000) | PRENATAL AND Slovakia AND (cohort OR study) |  | 64 | 2007-2020 |
| **SPATZ**[40] | 2012 | Ulm SPATZ health study[Title/Abstract] AND (cohort OR study) |  | 18 | 2014-2020 |
| **TEDS**[41] | 1994 | Twins early development study |  | 110 | 2002-2020 |
| **TERNEUZEN**[42] | 1977 | Terneuzen AND 1977 AND cohort |  | 1 | 2008 |
| **TURKU**[43] | 1981 | (Finnish OR Finland) AND 1981 Birth Cohort Study AND Turku |  | 17 | 1988-2020 |
| **UBCS**[44] | 2000 | Ubcs[Title/Abstract] AND Ulm |  | 9 | 2014-2020 |
| **WHISTLER**[45] | 2003 | WHISTLER AND Wheezing Illnesses Study in Leidsche Rijn AND Netherlands |  | 19 | 2004-2020 |
|  |  |  |  | 1512 |  |

**References**

1. Baxter-Jones AD, Cardy AH, Helms PJ, Phillips DO, Smith WC. Influence of socioeconomic conditions on growth in infancy: the 1921 Aberdeen birth cohort. Arch Dis Child. 1999;81:5–9.

2. Ludvigsson JF, Ludvigsson J. Coeliac disease in the father affects the newborn. Gut. 2001;49:169–75.

3. Doğruel D, Bingöl G, Yılmaz M, Altıntaş DU. The ADAPAR Birth Cohort Study: Food Allergy Results at Five Years and New Insights. Int Arch Allergy Immunol. 2016;169:57–61.

4. Patural H. Autonomic Nervous System: A Biomarker of Neurodevelopmental Comportment- the AuBE Study. J Clin Trials. 2014;04.

5. Lannerö E, Kull I, Wickman M, Pershagen G, Nordvall SL. Environmental risk factors for allergy and socioeconomic status in a birth cohort (BAMSE). Pediatr Allergy Immunol Off Publ Eur Soc Pediatr Allergy Immunol. 2002;13:182–7.

6. Skovgaard AM, Olsen EM, Houmann T, Christiansen E, Samberg V, Lichtenberg A, et al. The Copenhagen County child cohort: design of a longitudinal study of child mental health. Scand J Public Health. 2005;33:197–202.

7. Dejmek J, Solanský I, Benes I, Lenícek J, Srám RJ. The impact of polycyclic aromatic hydrocarbons and fine particles on pregnancy outcome. Environ Health Perspect. 2000;108:1159–64.

8. Kjaer HF, Eller E, Høst A, Andersen KE, Bindslev-Jensen C. The prevalence of allergic diseases in an unselected group of 6-year-old children. The DARC birth cohort study. Pediatr Allergy Immunol Off Publ Eur Soc Pediatr Allergy Immunol. 2008;19:737–45.

9. Kroke A, Manz F, Kersting M, Remer T, Sichert-Hellert W, Alexy U, et al. The DONALD Study. History, current status and future perspectives. Eur J Nutr. 2004;43:45–54.

10. Veldwijk J, Scholtens S, Hornstra G, Bemelmans WJE. Body mass index and cognitive ability of young children. Obes Facts. 2011;4:264–9.

11. Lødrup Carlsen KC. The environment and childhood asthma (ECA) study in Oslo: ECA-1 and ECA-2. Pediatr Allergy Immunol Off Publ Eur Soc Pediatr Allergy Immunol. 2002;13:29–31.

12. Vandentorren S, Bois C, Pirus C, Sarter H, Salines G, Leridon H, et al. Rationales, design and recruitment for the Elfe longitudinal study. BMC Pediatr. 2009;9:58.

13. Hogervorst JGF, Madhloum N, Saenen ND, Janssen BG, Penders J, Vanpoucke C, et al. Prenatal particulate air pollution exposure and cord blood homocysteine in newborns: Results from the ENVIRONAGE birth cohort. Environ Res. 2019;168:507–13.

14. Costeloe K, Hennessy E, Gibson AT, Marlow N, Wilkinson AR. The EPICure study: outcomes to discharge from hospital for infants born at the threshold of viability. Pediatrics. 2000;106:659–71.

15. Boudet-Berquier J, Salanave B, de Launay C, Castetbon K. Introduction of complementary foods with respect to French guidelines: description and associated socio-economic factors in a nationwide birth cohort (Epifane survey). Matern Child Nutr. 2017;13.

16. Center for Epidemiology and Biostatistics, INSERM. Epipage 2. https://epipage2.inserm.fr/index.php/en. Accessed 17 May 2022.

17. McBride D, Keil T, Grabenhenrich L, Dubakiene R, Drasutiene G, Fiocchi A, et al. The EuroPrevall birth cohort study on food allergy: baseline characteristics of 12,000 newborns and their families from nine European countries. Pediatr Allergy Immunol Off Publ Eur Soc Pediatr Allergy Immunol. 2012;23:230–9.

18. The David Hide Asthma & Allergy Research Centre, UK. Food Allergy and Intolerance Research (FAIR). David Hide Asthma & Allergy Research Centre. http://www.allergyresearch.org.uk/studies/food-allergy-intolerance-research-fair/. Accessed 17 May 2022.

19. Den Hond E, Govarts E, Bruckers L, Schoeters G. Determinants of polychlorinated aromatic hydrocarbons in serum in three age classes--Methodological implications for human biomonitoring. Environ Res. 2009;109:495–502.

20. Correia S, Rodrigues T, Barros H. Socioeconomic variations in female fertility impairment: a study in a cohort of Portuguese mothers. BMJ Open. 2014;4:e003985.

21. Porta D, Forastiere F, Di Lallo D, Perucci CA, Grupo Collaborativo GASPII. [Enrolment and follow-up of a birth cohort in Rome]. Epidemiol Prev. 2007;31:303–8.

22. van Jaarsveld CHM, Johnson L, Llewellyn C, Wardle J. Gemini: a UK twin birth cohort with a focus on early childhood weight trajectories, appetite and the family environment. Twin Res Hum Genet Off J Int Soc Twin Stud. 2010;13:72–8.

23. Heinrich J, Brüske I, Cramer C, Hoffmann U, Schnappinger M, Schaaf B, et al. GINIplus and LISAplus - Design and selected results of two German birth cohorts about natural course of atopic diseases and their determinants. Allergol Sel. 2017;1:85–95.

24. Parkinson KN, Pearce MS, Dale A, Reilly JJ, Drewett RF, Wright CM, et al. Cohort profile: the Gateshead Millennium Study. Int J Epidemiol. 2011;40:308–17.

25. Growing up in Scotland. https://growingupinscotland.org.uk/about-gus/study-design-and-methodology/. Accessed 17 May 2022.

26. Roswall J, Almqvist-Tangen G, Holmén A, Alm B, Bergman S, Dahlgren J, et al. Overweight at four years of age in a Swedish birth cohort: influence of neighbourhood-level purchasing power. BMC Public Health. 2016;16:546.

27. Eggesbø M, Stigum H, Longnecker MP, Polder A, Aldrin M, Basso O, et al. Levels of hexachlorobenzene (HCB) in breast milk in relation to birth weight in a Norwegian cohort. Environ Res. 2009;109:559–66.

28. Lanari M, Prinelli F, Adorni F, Di Santo S, Vandini S, Silvestri M, et al. Risk factors for bronchiolitis hospitalization during the first year of life in a multicenter Italian birth cohort. Ital J Pediatr. 2015;41:40.

29. Brandstetter S, Toncheva AA, Niggel J, Wolff C, Gran S, Seelbach-Göbel B, et al. KUNO-Kids birth cohort study: rationale, design, and cohort description. Mol Cell Pediatr. 2019;6:1.

30. Zutavern A, Rzehak P, Brockow I, Schaaf B, Bollrath C, von Berg A, et al. Day care in relation to respiratory-tract and gastrointestinal infections in a German birth cohort study. Acta Paediatr Oslo Nor 1992. 2007;96:1494–9.

31. Kuehni CE, Brooke AM, Strippoli M-PF, Spycher BD, Davis A, Silverman M. Cohort profile: the Leicester respiratory cohorts. Int J Epidemiol. 2007;36:977–85.

32. de Korte-de Boer D, Mommers M, Creemers HM, Dompeling E, Feron FJ, Gielkens-Sijstermans CM, et al. LucKi Birth Cohort Study: rationale and design. BMC Public Health. 2015;15:934.

33. Nickel R, Niggemann B, Grüber C, Kulig M, Wahn U, Lau S. How should a birth cohort study be organised? Experience from the German MAS cohort study. Paediatr Respir Rev. 2002;3:169–76.

34. Brescianini S, Fagnani C, Toccaceli V, Medda E, Nisticò L, D’Ippolito C, et al. An update on the Italian Twin Register: advances in cohort recruitment, project building and network development. Twin Res Hum Genet Off J Int Soc Twin Stud. 2013;16:190–6.

35. Paananen R, Gissler M. Cohort profile: the 1987 Finnish Birth Cohort. Int J Epidemiol. 2012;41:941–5.

36. Clarisse B, Nikasinovic L, Poinsard R, Just J, Momas I. The Paris prospective birth cohort study: which design and who participates? Eur J Epidemiol. 2007;22:203–10.

37. Jusko TA, De Roos AJ, Schwartz SM, Lawrence BP, Palkovicova L, Nemessanyi T, et al. A cohort study of developmental polychlorinated biphenyl (PCB) exposure in relation to post-vaccination antibody response at 6-months of age. Environ Res. 2010;110:388–95.

38. Farchi S, Forastiere F, Vecchi Brumatti L, Alviti S, Arnofi A, Bernardini T, et al. Piccolipiù, a multicenter birth cohort in Italy: protocol of the study. BMC Pediatr. 2014;14:36.

39. Dunlop AL, Reichrtova E, Palcovicova L, Ciznar P, Adamcakova-Dodd A, Smith SJ, et al. Environmental and dietary risk factors for infantile atopic eczema among a Slovak birth cohort. Pediatr Allergy Immunol Off Publ Eur Soc Pediatr Allergy Immunol. 2006;17:103–11.

40. Braig S, Weiss JM, Stalder T, Kirschbaum C, Rothenbacher D, Genuneit J. Maternal prenatal stress and child atopic dermatitis up to age 2 years: The Ulm SPATZ health study. Pediatr Allergy Immunol Off Publ Eur Soc Pediatr Allergy Immunol. 2017;28:144–51.

41. TEDS – TWINS Early Development Study. https://www.ukri.org/councils/mrc/facilities-and-resources/find-an-mrc-facility-or-resource/cohort-directory/. Accessed 17 May 2022.

42. de Kroon MLA. The Terneuzen Birth Cohort: Detection and Prevention of Overweight and Cardiometabolic Risk from Infancy Onward. PhD-Thesis - Research and graduation internal. 2011.

43. Piekkala P, Kero P, Sillanpää M, Erkkola R. Growth and development of infants surviving respiratory distress syndrome: a 2-year follow-up. Pediatrics. 1987;79:529–37.

44. Christiansen H, Brandt S, Walter V, Wabitsch M, Rothenbacher D, Brenner H, et al. Prediction of BMI at age 11 in a longitudinal sample of the Ulm Birth Cohort Study. PloS One. 2017;12:e0182338.

45. Katier N, Uiterwaal CSPM, de Jong BM, Kimpen JLL, Verheij TJ, Grobbee DE, et al. The Wheezing Illnesses Study Leidsche Rijn (WHISTLER): rationale and design. Eur J Epidemiol. 2004;19:895–903.
