# Supplementary material for: European birth cohorts: a consideration of what they have addressed so far
Source: BMC Pediatr. 2022 Sep 15;22:548. doi: 10.1186/s12887-022-03599-2 (PMC9476293; doi:10.1186/s12887-022-03599-2)
Supplement: Supplementary file 2 — Additional file 2. [file 12887_2022_3599_MOESM2_ESM.docx]

**Additional file 2.** Weights of the connections
