# Supplementary figures and images for: European birth cohorts: a consideration of what they have addressed so far
Source: BMC Pediatr. 2022 Sep 15;22:548. doi: 10.1186/s12887-022-03599-2 (PMC9476293; doi:10.1186/s12887-022-03599-2)

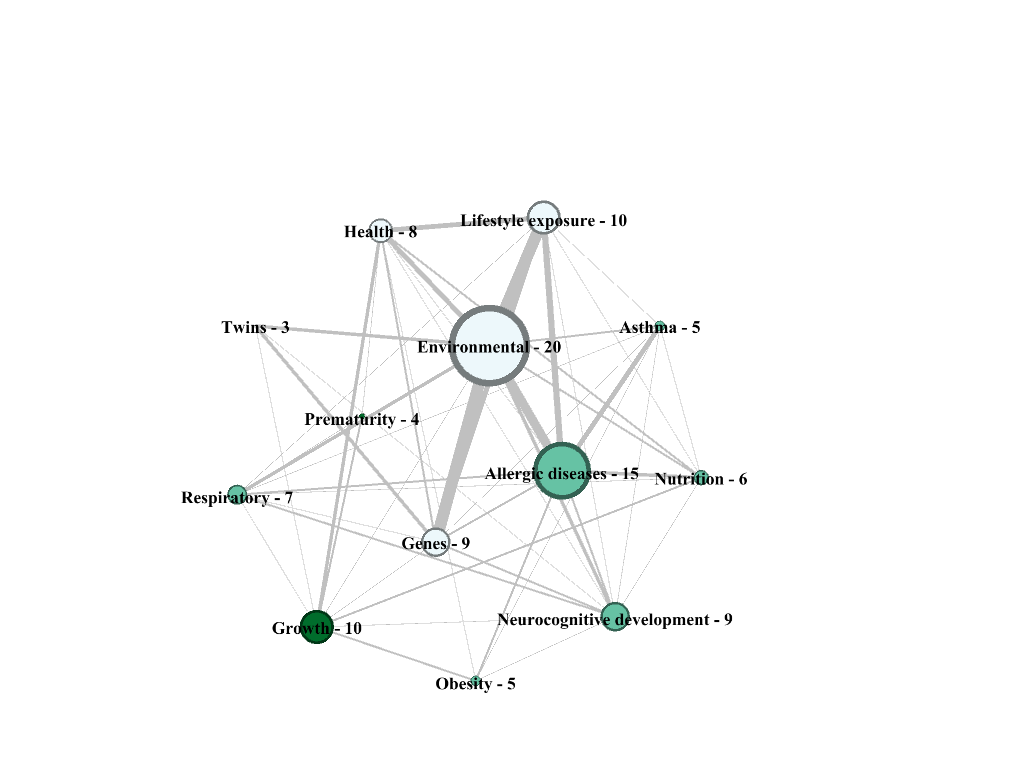

Supplement: Supplementary file 3 — Additional file 3. [file 12887_2022_3599_MOESM3_ESM.png]
