# Supplementary material for: European birth cohorts: a consideration of what they have addressed so far
Source: BMC Pediatr. 2022 Sep 15;22:548. doi: 10.1186/s12887-022-03599-2 (PMC9476293; doi:10.1186/s12887-022-03599-2)
Supplement: Supplementary file 5 — Additional file 5. [file 12887_2022_3599_MOESM5_ESM.docx]

**Additional file 5.** Publications indexed with the keyword "Smoking" from the Environmental (12/20 cohorts, 35 publications) and Prematurity cohorts (3/4 cohorts, 22 publications), and their main topics.

| Cohort | Pub date | Citation number | Topic | Smoking in adolescence directly (as opposed to parental smoking) |
| --- | --- | --- | --- | --- |
| Environmental cohorts |  |  |  |  |
| ABIS | 2004 | [1] | Attitudes |  |
| ABIS | 2008 | [2] | Breastfeeding |  |
| ABIS | 2005 | [3] | Breastfeeding |  |
| ABIS | 2005 | [4] | Coeliac disease |  |
| ABIS | 2006 | [5] | Constipations/gastrointestinal symptoms |  |
| ABIS | 2008 | [6] | Diabetes |  |
| ABIS | 2007 | [7] | High sugar intake |  |
| ABIS | 2002 | [8] | Psychological mechanisms and development of diabetes |  |
| ELFE | 2018 | [9] | Migrant origin |  |
| ELFE | 2015 | [10] | Migrant status |  |
| ELFE | 2015 | [11] | Psychological distress |  |
| ELFE | 2017 | [12] | Risk factors for parental smoking |  |
| ELFE | 2018 | [13] | Wheezing |  |
| ENVIRONAGE | 2017 | [14] | Cord plasma insulin (Smoking as confounder) |  |
| ENVIRONAGE | 2017 | [15] | Low birth weight (epigenetics and mitochondrial function in foetal tissues) |  |
| ENVIRONAGE | 2016 | [16] | Oxidative stress during foetal life |  |
| ENVIRONAGE | 2020 | [17] | Placental mtDNA methylation |  |
| ENVIRONAGE | 2020 | [18] | Placental transfer of glycidol |  |
| FLEHS-I | 2017 | [19] | exposure biomarkers (incl. smoke-related one) in people of diff ages |  |
| GASPII | 2007 | [20] | Attitude towards cohort participation |  |
| HUMIS | 2009 | [21] | Birth weight |  |
| LISAPLUS | 2011 | [22] | Behaviour problems |  |
| LISAPLUS | 2012 | [23] | Healthcare costs (second-hand smoke) |  |
| LISAPLUS | 2011 | [24] | Insulin resistance |  |
| LISAPLUS | 2016 | [25] | Lung health |  |
| LISAPLUS | 2017 | [26] | Lung health |  |
| LISAPLUS | 2013 | [27] | Overweight |  |
| LISAPLUS | 2012 | [28] | Oxidative stress and gene influence on obstructive bronchitis |  |
| LISAPLUS | 2017 | [29] | Systemic inflammatory markers | YES |
| MAS-90 | 2008 | [30] | Fat mass development |  |
| MUBICOS | 2018 | [31] | Body mass index | YES |
| PARISCOHORT | 2016 | [32] | Wheeze |  |
| PRENATAL COHORT | 2008 | [33] | PCBs and birth weight (Smoking as confounder) |  |
| PRENATAL COHORT | 2018 | [34] | Synergy with endocrine disruption chemicals |  |
| WHISTLER | 2006 | [35] | Passive respiratory mechanics (Smoking as covariate) |  |
| Prematurity cohorts |  |  |  |  |
| EPICURE1995 | 2008 | [36] | Later respiratory health in preterms (Smoking as covariate) |  |
| ITALNEONAT | 2015 | [37] | Bronchiolitis |  |
| NFBC1986 | 2016 | [38] | Aerobic fitness |  |
| NFBC1986 | 2019 | [39] | Body mass index | YES |
| NFBC1986 | 2013 | [40] | Body mass index and lumbar disc degeneration | YES |
| NFBC1986 | 1997 | [41] | Ecological and individual predictors of birthweight (Smoking as covariate) |  |
| NFBC1986 | 2014 | [42] | Lifestyle and psychosocial problems | YES |
| NFBC1986 | 2016 | [43] | Low back pain | YES |
| NFBC1986 | 2008 | [44] | Low back pain | YES |
| NFBC1986 | 2019 | [45] | Musculoskeletal pain | YES |
| NFBC1986 | 2017 | [46] | Musculoskeletal pain |  |
| NFBC1986 | 2014 | [47] | Musculoskeletal pain | YES |
| NFBC1986 | 2010 | [48] | Musculoskeletal pain | YES |
| NFBC1986 | 2010 | [49] | Musculoskeletal pain | YES |
| NFBC1986 | 2017 | [50] | Physical activity and cardiorespiratory fitness levels |  |
| NFBC1986 | 2012 | [51] | Pre-pregnancy obesity and asthma symptoms in adolescents (Smoking as confounder) |  |
| NFBC1986 | 2018 | [52] | Psychosis | YES |
| NFBC1986 | 2017 | [53] | Risk of smoking |  |
| NFBC1986 | 2016 | [54] | Risk of substance use, conduct problems, and risky sex |  |
| NFBC1986 | 2013 | [55] | Sciatica pain | YES |
| NFBC1986 | 2010 | [56] | Systemic inflammation | YES |
| NFBC1986 | 2012 | [57] | Thyroid hormone levels |  |

**References**

1. Johansson A, Hermansson G, Ludvigsson J. Parents’ attitudes to children’s tobacco smoke exposure and how the issue is handled in health care. J Pediatr Health Care. 2004;18:228–35.

2. Huus K, Ludvigsson JF, Enskär K, Ludvigsson J. Exclusive breastfeeding of Swedish children and its possible influence on the development of obesity: a prospective cohort study. BMC Pediatr. 2008;8:42.

3. Ludvigsson JF, Ludvigsson J. Socio-economic determinants, maternal smoking and coffee consumption, and exclusive breastfeeding in 10 205 children: Socio-economic factors and exclusive breastfeeding. Acta Paediatr. 2007;94:1310–9.

4. Ludvigsson JF, Ludvigsson J. Parental smoking and risk of coeliac disease in offspring. Scand J Gastroenterol. 2005;40:336–42.

5. Ludvigsson JF, Group FTAS. Epidemiological study of constipation and other gastrointestinal symptoms in 8000 children. Acta Paediatr. 2006;95:573–80.

6. Johansson A, Hermansson G, Ludvigsson J, for the ABIS Study Group. Tobacco Exposure and Diabetes-Related Autoantibodies in Children. Ann N Y Acad Sci. 2008;1150:197–9.

7. Brekke HK, van Odijk J, Ludvigsson J. Predictors and dietary consequences of frequent intake of high-sugar, low-nutrient foods in 1-year-old children participating in the ABIS study. Br J Nutr. 2007;97:176–81.

8. Sepa A, Frodi A, Ludvigsson J. Could Parenting Stress and Lack of Support/Confidence Function as Mediating Mechanisms between Certain Environmental Factors and the Development of Autoimmunity in Children?: A Study within ABIS. Ann N Y Acad Sci. 2006;958:431–5.

9. El-Khoury Lesueur F, Sutter-Dallay A-L, Panico L, Azria E, Van der Waerden J, Regnault Vauvillier N, et al. The perinatal health of immigrant women in France: a nationally representative study. Int J Public Health. 2018;63:1027–36.

10. Melchior M, Chollet A, Glangeaud-Freudenthal N, Saurel-Cubizolles M-J, Dufourg M-N, van der Waerden J, et al. Tobacco and alcohol use in pregnancy in France: The role of migrant status. Addict Behav. 2015;51:65–71.

11. Bales M, Pambrun E, Melchior M, Glangeaud-Freudenthal NM-C, Charles M-A, Verdoux H, et al. Prenatal Psychological Distress and Access to Mental Health Care in the ELFE Cohort. Eur Psychiatry. 2015;30:322–8.

12. El-Khoury F, Sutter-Dallay A-L, Van Der Waerden J, Surkan P, Martins S, Keyes K, et al. Smoking Trajectories during the Perinatal Period and Their Risk Factors: The Nationally Representative French ELFE (Etude Longitudinale Française Depuis l’Enfance) Birth Cohort Study. Eur Addict Res. 2017;23:194–203.

13. Hallit S, Leynaert B, Delmas MC, Rocchi S, De Blic J, Marguet C, et al. Wheezing phenotypes and risk factors in early life: The ELFE cohort. PLOS ONE. 2018;13:e0196711.

14. Madhloum N, Janssen BG, Martens DS, Saenen ND, Bijnens E, Gyselaers W, et al. Cord plasma insulin and in utero exposure to ambient air pollution. Environ Int. 2017;105:126–32.

15. Janssen BG, Gyselaers W, Byun H-M, Roels HA, Cuypers A, Baccarelli AA, et al. Placental mitochondrial DNA and CYP1A1 gene methylation as molecular signatures for tobacco smoke exposure in pregnant women and the relevance for birth weight. J Transl Med. 2017;15:5.

16. Saenen ND, Vrijens K, Janssen BG, Madhloum N, Peusens M, Gyselaers W, et al. Placental Nitrosative Stress and Exposure to Ambient Air Pollution During Gestation: A Population Study. Am J Epidemiol. 2016;184:442–9.

17. Vos S, Nawrot TS, Martens DS, Byun H-M, Janssen BG. Mitochondrial DNA methylation in placental tissue: a proof of concept study by means of prenatal environmental stressors. Epigenetics. 2021;16:121–31.

18. Monien BH, Abraham K, Nawrot TS, Hogervorst JGF. Levels of the hemoglobin adduct N-(2,3-Dihydroxypropyl)-valine in cord and maternal blood: Prenatal transfer of glycidol in the ENVIRONAGE birth cohort. Toxicol Lett. 2020;332:82–7.

19. Schoeters G, Govarts E, Bruckers L, Den Hond E, Nelen V, De Henauw S, et al. Three cycles of human biomonitoring in Flanders − Time trends observed in the Flemish Environment and Health Study. Int J Hyg Environ Health. 2017;220:36–45.

20. Porta D, Forastiere F, Di Lallo D, Perucci CA, Grupo Collaborativo GASPII. [Enrolment and follow-up of a birth cohort in Rome]. Epidemiol Prev. 2007;31:303–8.

21. Eggesbø M, Stigum H, Longnecker MP, Polder A, Aldrin M, Basso O, et al. Levels of hexachlorobenzene (HCB) in breast milk in relation to birth weight in a Norwegian cohort. Environ Res. 2009;109:559–66.

22. Tiesler CMT, Chen C-M, Sausenthaler S, Herbarth O, Lehmann I, Schaaf B, et al. Passive smoking and behavioural problems in children: Results from the LISAplus prospective birth cohort study. Environ Res. 2011;111:1173–9.

23. Batscheider A, Zakrzewska S, Heinrich J, Teuner CM, Menn P, Bauer CP, et al. Exposure to second-hand smoke and direct healthcare costs in children – results from two German birth cohorts, GINIplus and LISAplus. BMC Health Serv Res. 2012;12:344.

24. Thiering E, Brüske I, Kratzsch J, Thiery J, Sausenthaler S, Meisinger C, et al. Prenatal and postnatal tobacco smoke exposure and development of insulin resistance in 10 year old children. Int J Hyg Environ Health. 2011;214:361–8.

25. Smith MP, von Berg A, Berdel D, Bauer C-P, Hoffmann B, Koletzko S, et al. Physical activity is not associated with spirometric indices in lung-healthy German youth. Eur Respir J. 2016;48:428–40.

26. Luzak A, Fuertes E, Flexeder C, Standl M, von Berg A, Berdel D, et al. Which early life events or current environmental and lifestyle factors influence lung function in adolescents? – results from the GINIplus & LISAplus studies. Respir Res. 2017;18:138.

27. for the GINIplus and LISAplus Study Group, Pei Z, Flexeder C, Fuertes E, Thiering E, Koletzko B, et al. Early life risk factors of being overweight at 10 years of age: results of the German birth cohorts GINIplus and LISAplus. Eur J Clin Nutr. 2013;67:855–62.

28. Bauer M, Gräbsch C, Schlink U, Klopp N, Illig T, Krämer U, et al. Genetic association between obstructive bronchitis and enzymes of oxidative stress. Metabolism. 2012;61:1771–9.

29. Pitchika V, Thiering E, Metz I, Rothmaier K, Willenberg A, Hickel R, et al. Gingivitis and lifestyle influences on high-sensitivity C-reactive protein and interleukin 6 in adolescents. J Clin Periodontol. 2017;44:372–81.

30. Karaolis-Danckert N, Buyken AE, Kulig M, Kroke A, Forster J, Kamin W, et al. How pre- and postnatal risk factors modify the effect of rapid weight gain in infancy and early childhood on subsequent fat mass development: results from the Multicenter Allergy Study 90. Am J Clin Nutr. 2008;87:1356–64.

31. Piirtola M, Jelenkovic A, Latvala A, Sund R, Honda C, Inui F, et al. Association of current and former smoking with body mass index: A study of smoking discordant twin pairs from 21 twin cohorts. PloS One. 2018;13:e0200140.

32. Vardavas CI, Hohmann C, Patelarou E, Martinez D, Henderson AJ, Granell R, et al. The independent role of prenatal and postnatal exposure to active and passive smoking on the development of early wheeze in children. Eur Respir J. 2016;48:115–24.

33. Sonneborn D, Park H-Y, Petrik J, Kocan A, Palkovicova L, Trnovec T, et al. Prenatal polychlorinated biphenyl exposures in eastern Slovakia modify effects of social factors on birthweight. Paediatr Perinat Epidemiol. 2008;22:202–13.

34. Govarts E, Iszatt N, Trnovec T, de Cock M, Eggesbø M, Palkovicova Murinova L, et al. Prenatal exposure to endocrine disrupting chemicals and risk of being born small for gestational age: Pooled analysis of seven European birth cohorts. Environ Int. 2018;115:267–78.

35. Katier N, Uiterwaal CSPM, de Jong BM, Verheij TJM, van der Ent CK, for the WHISTLER study group. Passive respiratory mechanics measured during natural sleep in healthy term neonates and infants up to 8 weeks of life. Pediatr Pulmonol. 2006;41:1058–64.

36. Hennessy EM, Bracewell MA, Wood N, Wolke D, Costeloe K, Gibson A, et al. Respiratory health in pre-school and school age children following extremely preterm birth. Arch Dis Child. 2008;93:1037–43.

37. Lanari M, Vandini S, Adorni F, Prinelli F, Di Santo S, on behalf of the “Study Group of Italian Society of Neonatology on Risk Factors for RSV Hospitalization,” et al. Prenatal tobacco smoke exposure increases hospitalizations for bronchiolitis in infants. Respir Res. 2015;16:152.

38. Hagnäs M, Cederberg H, Jokelainen J, Mikkola I, Rajala U, Keinänen-Kiukaanniemi S. Association of maternal smoking during pregnancy with aerobic fitness of offspring in young adulthood: a prospective cohort study. BJOG Int J Obstet Gynaecol. 2016;123:1789–95.

39. Marttila-Tornio K, Ruotsalainen H, Miettunen J, Männikkö N, Kääriäinen M. Clusters of health behaviours and their relation to body mass index among adolescents in Northern Finland. Scand J Caring Sci. 2020;34:666–74.

40. Takatalo J, Karppinen J, Taimela S, Niinimäki J, Laitinen J, Blanco Sequeiros R, et al. Body mass index is associated with lumbar disc degeneration in young Finnish males: subsample of Northern Finland birth cohort study 1986. BMC Musculoskelet Disord. 2013;14:87.

41. Järvelin MR, Elliott P, Kleinschmidt I, Martuzzi M, Grundy C, Hartikainen AL, et al. Ecological and individual predictors of birthweight in a northern Finland birth cohort 1986. Paediatr Perinat Epidemiol. 1997;11:298–312.

42. Heikkala E, Remes J, Paananen M, Taimela S, Auvinen J, Karppinen J. Accumulation of lifestyle and psychosocial problems and persistence of adverse lifestyle over two-year follow-up among Finnish adolescents. BMC Public Health. 2014;14:542.

43. Mikkonen P, Heikkala E, Paananen M, Remes J, Taimela S, Auvinen J, et al. Accumulation of psychosocial and lifestyle factors and risk of low back pain in adolescence: a cohort study. Eur Spine J. 2016;25:635–42.

44. Mikkonen P, Leino-Arjas P, Remes J, Zitting P, Taimela S, Karppinen J. Is Smoking a Risk Factor for Low Back Pain in Adolescents?: A Prospective Cohort Study: Spine. 2008;33:527–32.

45. Heikkala E, Paananen M, Taimela S, Auvinen J, Karppinen J. Associations of co‐occurring psychosocial and lifestyle factors with multisite musculoskeletal pain during late adolescence—A birth cohort study. Eur J Pain. 2019;23:1486–96.

46. Määttä A-J, Paananen M, Marttila R, Auvinen J, Miettunen J, Karppinen J. Maternal Smoking During Pregnancy Is Associated With Offspring’s Musculoskeletal Pain in Adolescence: Structural Equation Modeling. Nicotine Tob Res. 2016;:ntw325.

47. Jussila L, Paananen M, Näyhä S, Taimela S, Tammelin T, Auvinen J, et al. Psychosocial and lifestyle correlates of musculoskeletal pain patterns in adolescence: a 2-year follow-up study. Eur J Pain Lond Engl. 2014;18:139–46.

48. Paananen MV, Auvinen JP, Taimela SP, Tammelin TH, Kantomaa MT, Ebeling HE, et al. Psychosocial, mechanical, and metabolic factors in adolescents’ musculoskeletal pain in multiple locations: A cross-sectional study. Eur J Pain. 2010;14:395–401.

49. Paananen MV, Taimela SP, Auvinen JP, Tammelin TH, Kantomaa MT, Ebeling HE, et al. Risk factors for persistence of multiple musculoskeletal pains in adolescence: A 2-year follow-up study. Eur J Pain. 2010;14:1026–32.

50. Tikanmäki M, Tammelin T, Vääräsmäki M, Sipola-Leppänen M, Miettola S, Pouta A, et al. Prenatal determinants of physical activity and cardiorespiratory fitness in adolescence – Northern Finland Birth Cohort 1986 study. BMC Public Health. 2017;17:346.

51. Patel SP, Rodriguez A, Little MP, Elliott P, Pekkanen J, Hartikainen A-L, et al. Associations between pre-pregnancy obesity and asthma symptoms in adolescents. J Epidemiol Community Health. 2012;66:809–14.

52. Mustonen A, Ahokas T, Nordström T, Murray GK, Mäki P, Jääskeläinen E, et al. Smokin‘ hot: adolescent smoking and the risk of psychosis. Acta Psychiatr Scand. 2018;138:5–14.

53. Niemelä S, Räisänen A, Koskela J, Taanila A, Miettunen J, Ramsay H, et al. The effect of prenatal smoking exposure on daily smoking among teenage offspring: Prenatal smoking exposure and offspring smoking. Addiction. 2017;112:134–43.

54. Mason WA, January S-AA, Chmelka MB, Parra GR, Savolainen J, Miettunen J, et al. Cumulative contextual risk at birth in relation to adolescent substance use, conduct problems, and risky sex: General and specific predictive associations in a Finnish birth cohort. Addict Behav. 2016;58:161–6.

55. Karjalainen U, Paananen M, Okuloff A, Taimela S, Auvinen J, Männikkö M, et al. Role of Environmental Factors and History of Low Back Pain in Sciatica Symptoms Among Finnish Adolescents: Spine. 2013;38:1105–11.

56. Pirkola J, Vääräsmäki M, Ala-Korpela M, Bloigu A, Canoy D, Hartikainen A-L, et al. Low-grade, systemic inflammation in adolescents: association with early-life factors, gender, and lifestyle. Am J Epidemiol. 2010;171:72–82.

57. Männistö T, Hartikainen A-L, Vääräsmäki M, Bloigu A, Surcel H-M, Pouta A, et al. Smoking and Early Pregnancy Thyroid Hormone and Anti-Thyroid Antibody Levels in Euthyroid Mothers of the Northern Finland Birth Cohort 1986. Thyroid. 2012;22:944–50.
