# Supplementary material for: European birth cohorts: a consideration of what they have addressed so far
Source: BMC Pediatr. 2022 Sep 15;22:548. doi: 10.1186/s12887-022-03599-2 (PMC9476293; doi:10.1186/s12887-022-03599-2)
Supplement: Supplementary file 7 — Additional file 7. [file 12887_2022_3599_MOESM7_ESM.docx]

**Additional material**:

**Additional file 1**: Search strings used to find the cohorts’ publications in PubMed (.docx);

**Additional file 2**: Weights of the connections (.docx);

**Additional file 3**: Community detection analysis, showing 3 clusters (3 different shades of green) (.tif);

**Additional file 4**: Centrality measures (docx);

**Additional file 5**: Publications on smoking (docx);

**Additional file 6** - PRISMA-ScR-Checklist (pdf);

**Additional file 7** - Legends(docx).
